# The West Texas Medical Society

**Published:** 1898-12

**Authors:** 


THE WEST TEXAS MEDICAL SOCIETY.—We produced in our last issue two notable papers, read before this Association at its recent meeting; one by Dr. F. Paschall, one by Dr. A. C. McDaniel. We are pleased to note the continued prosperity of this organization and the unabated interest in its meetings. It has been suggested that the West Texas Medical Society and the Austin District Medical Society unite in one organization and meet alternately at Austin and San Antonio. Interest is always greater where there is a large attendance. Who will move to unite them?

---

DR. DANIEL DU PRE died at his home in Dallas, Texas, November 28, aged 76 years. Dr. Du Pre was a native of Tennessee, and a graduate of the Pennsylvania Medical College. He was a distinguished Confederate surgeon, and served at one time as Brigade Surgeon on the staff of Gen. Dan Adams, of Mississippi, and later in the hospitals of Georgia, during the late War of Secession. He was a man of splendid physique, and of fine literary and professional attainments, much respected by his colleagues and the community where he spent many years in the active practice of his profession. His loss will be much felt.

